# Endocrine toxicity of immune checkpoint inhibitors: a real-world study leveraging US Food and Drug Administration adverse events reporting system

**DOI:** 10.1186/s40425-019-0754-2

**Published:** 2019-11-06

**Authors:** Yinghong Zhai, Xiaofei Ye, Fangyuan Hu, Jinfang Xu, Xiaojing Guo, Yonglong Zhuang, Jia He

**Affiliations:** 1 Tongji University School of Medicine, 1239 Siping Road, Yangpu District, Shanghai, 200092 China; 2 Department of Health Statistics, Second Military Medical University, No. 800 Xiangyin Road, Shanghai, 200433 China; 3 Beijing Bioknow Information Technology Co.Ltd., Beijing, China

**Keywords:** Immune checkpoint inhibitors, Endocrine toxicities, FAERS, PD-1/PD-L1, CTLA-4, Monotherapy, Combination therapy

## Abstract

**Background:**

Immune-checkpoint inhibitors (ICIs) emerged as a novel class of drugs for the treatment of a broad spectrum of malignancies. ICIs can produce durable antitumor responses but they are also associated with immune-related adverse events (irAEs). Endocrinopathies have reported as one of the most common irAEs of ICIs.

**Methods:**

This study aimed to quantify association of endocrine adverse events (AEs) and ICI therapy and also to characterize the profiles of ICI-related endocrine complications from real-world practice. Data from the first quarter of 2014 to first quarter of 2019 in FDA Adverse Event Reporting System (FAERS) database were gathered to conduct disproportionality analysis. The definition of endocrine AEs relied on the preferred terms (PTs) provided by the Medical Dictionary for Regulatory Activities (MedDRA). Two signal indices based on statistical shrinkage transformation, reporting odds ratios (ROR) and information component (IC), were used to evaluate correlations between ICIs and endocrine events. For ROR, it was defined a signal if the lower limit of the 95% confidence interval (ROR_025_) more than one, with at least 3 cases. For IC, lower end of the 95% confidence interval of IC (IC_025_) exceeding zero was deemed statistically significant.

**Results:**

A total of 29,294,336 records were involved, among these 6260 records related to endocrine AEs after ICIs treatment were identified. In general, male had a slightly lower reporting frequencies for ICIs-related endocrinopathies compared with female but not significant (ROR = 0.98 95%CI: 0.93–1.04) and the difference varied in several common endocrine AEs. Notably, in general, ICI drugs were significantly associated with over-reporting frequencies of endocrine complications, corresponding to IC_025_ = 2.49 and ROR_025_ = 5.99. For monotherapy, three strategies (anti-PD-1, anti-PD-L1 and anti-CTLA-4) were all associated with significant increasing endocrine events. Different reporting frequencies emerged when anti-CTLA-4 therapy was compared with anti-PD-1/PD-L1 medications for endocrine toxicities, corresponding to ROR = 1.68 (95%CI 1.55–1.83), ROR = 2.54 (95%CI 2.20–2.93), respectively. Combination therapy was associated with higher risk of endocrinopathies compared with monotherapy (ROR = 2.00, 95%CI 1.89–2.11). When further analysis, the spectrum of endocrine AEs differed in immunotherapy regimens. Hypothyroidism (*N* = 885,14.14%), adrenal insufficiency(*N* = 730,11.66%), hypophysitis (*N* = 688,10.99%) and hyperthyroidism (*N* = 472,7.54%) were top 4 ranked endocrine events after ICI therapy and their reporting frequency also differed in ICI immunotherapies.

**Conclusion:**

Our pharmacovigilance analysis shows a high reporting frequency of endocrine AEs provoked by ICI monotherapy (especially anti-CTLA-4 therapy) and further reinforced by combination therapy. In addition, treatment with different ICI immunotherapies may result in a unique and distinct profile of endocrinopathies. Early recognition and management of ICI-related endocrine irAEs is of vital importance in clinical practice.

## Introduction

Immune checkpoint inhibitors (ICIs) are a novel class of medications in cancer treatment and have rapidly gained popularity for their success in improving clinical outcomes in multiple cancer types [1]. Currently, ICIs include agents target programmed death-1 receptor (PD-1;nivolumab, pembrolizumab, cemiplimab), programmed death-ligand 1 (PD-L1;atezolizumab, avelumab, durvalumab), and cytotoxic T-lymphocyte-associated protein 4 (CTLA-4; ipilimumab, tremelimumab) [2].

The administration of ICIs, whereas, carry the risk of developing immune-related adverse events (irAEs) and may lead to serious and even fatal events [3, 4]. Endocrinopathies are among the most common irAEs associated with ICIs therapy including hypophysitis, thyroid dysfunction (hypothyroidism/hyperthyroidism), insulin-deficient diabetes mellitus [5].

Given the widespread use of ICIs in clinical practice and the potentially life-threatening nature of ICI-associated endocrinopathies if not promptly recognized and treated, it is critical for clinicians to realize the clinical manifestations and management of endocrinopathies triggered by ICIs. In the study, we conducted a disproportionality analysis leveraging a large pharmacovigilance database (FAERS) to characterize and evaluate endocrine toxicity associated with ICI regimens. While pharmacovigilance data may lack detailed clinical information, using this approach may help discovery potential drug-toxicity associations [6].

## Methods

### Study design and data sources

This retrospective, pharmacovigilance study is a disproportionality analysis based on FAERS database. FAERS is a collection of reports of AEs by consumers, healthcare providers, drug manufacturers, and others. It allows for the signal detection and quantification of the association between drugs and reporting of AEs [7]. Input data for this study were taken from the public release of the FAERS database, covering the period from the first quarter of 2014 through the first quarter of 2019.

### Procedures

Study drugs in this study included antibodies targeting PD-1 (nivolumab and pembrolizumab), PD-L1 (atezolizumab, avelumab, durvalumab), and CTLA-4 (ipilimumab, tremelimumab). Since the FAERS does not use a uniform coding system for medications, brand names and generic names were used to identify ICIs associated records. Severe patient outcomes were defined as life-threatening events or those causing death, hospitalization, disability, congenital anomaly, required intervention to prevent permanent impairment/damage or other significant medically important condition.

This study included all endocrine disorders (medDRA code 10014698) according to MedDRA *version 20.0*. In the FAERS database, each report is coded using PTs from MedDRA, the international medical terminology developed by the International Council for Harmonisation of Technical Requirements for Registration of Pharmaceuticals for Human Use.

### Statistical analysis

In pharmacovigilance study, disproportionality emerges when a specific adverse event is associated with a given drug [8]. Two data mining methods using proportional reports reporting odds ratio (ROR) and Bayesian confidence propagation neural networks of information components (IC) were used to calculate disproportionality [9, 10]. Statistical shrinkage transformation was applied to obtain robust results [11]. Shrinkage transformations statistical formula going as follows:

$$ \mathrm{ROR}=\frac{N_{observed}+0.5}{N_{expected}+0.5} $$

$$ \mathrm{IC}={\log}_2\frac{N_{observed}+0.5}{N_{expected}+0.5} $$

$$ {N}_{expected}=\frac{n_{drug}\ast {n}_{event}}{n_{total}} $$

*N*_*expected*_: the number of records expected for the selected drug-adverse event combination.

*N*_*observed*_: the observed number of records for the selected drug-adverse event combination.

*N*_*drug*_: the total number of records for the selected drug.

*N*_*event*_: the total number of total records for the selected adverse event.

*N*_*total*_: the total number of records in the database.

The calculation for ROR and IC employing two-by-two contingency tables of reported event counts for specific drug and other drugs. Disproportionality can be either calculated by the IC or reporting ROR when using full database as comparator, and only ROR when compared different drug strategies. For ROR, it was defined significant signal if the lower limit of the 95% confidence interval (ROR_025_) exceeded 1, with at least 3 cases. IC_025_ is the lower end of a 95% confidence interval for the IC and IC_025_ greater than 0 is the traditional threshold used in statistical signal detection at the Uppsala Monitoring Centre. All the analysis was performed with SAS version 9.4(SAS Institute Inc., Cary, NC, USA).

## Results

### Descriptive analysis

A total of 29,294,336 records were involved in the full FAERS dataset, among these 6260 were reported for endocrine AEs after ICIs treatment. The clinical characteristics of patients with ICIs induced endocrine toxicity were described in Table 1. Most cases were reported in 2016–2019, reflecting the substantially increased usage of ICIs recent years. Among all endocrinopathies, men accounted for a larger proportion than women regardless in ICIs (54.76% vs 33.47%) or any other drugs (40.90% vs 13.40%).Whereas, when further analysis, male had a slightly lower reporting frequencies for ICIs-related endocrinopathies compared with female but not significant (ROR = 0.98 95%CI: 0.93–1.04) and the difference varied in several common endocrine AEs (Additional file 1:Table S1;Figure S1). Hospitalization and other serious important medical events were the most frequently reported severe outcomes. Death or life-threatening events occurring in 1075(17.17%) endocrine AEs for ICIs indicating potentially life-threatening nature of ICI-related endocrinopathies.

**Table 1:** Clinical characteristics of patients with ICIs induced endocrine toxicity

	Endocrine AEs in ICIs (6260)	Endocrine AEs in other drugs (233338)
Gender		
Male	3428(54.76)	106,643(45.70)
Female	2095(33.47)	95,425(40.90)
Missing	737(11.77)	31,270(13.40)
Age		
< 65	2481(39.63)	91,368(39.16)
> =65	2496(39.87)	55,099(23.61)
Missing	1283(20.50)	86,871(37.23)
Year		
2014	116(1.85)	17,308(7.42)
2015	59(0.94)	37,813(16.21)
2016	944(15.08)	47,013(20.15)
2017	1572(25.11)	62,480(26.78)
2018	2838 (45.34)	55,743(23.89)
2019Q1	731(11.68)	12,981(5.56)
Outcome		
Death	601(9.60)	9752(4.18)
Life-threatening	474(7.57)	10,435(4.47)
Disability	108(1.73)	4958(2.12)
Hospitalization	2329(37.20)	69,842(29.93)
Congenital anomaly	0(0.00)	102(0.04)
Other serious	1605(25.64)	78,742(33.75)
Required intervention	1(0.02)	50(0.02)
Missing	1142(18.24)	59,457(25.48)
Report countries		
United States	1997(31.90)	124,384(53.31)
Japan	1748(27.92)	14,146(6.06)
Great Britain	181(2.89)	11,376(4.88)
France	393(6.28)	10,258(4.40)
Canada	62(0.99)	8741(3.75)
Italy	116(1.85)	7922(3.40)
Other countries	1033(16.50)	43,567(18.67)
Missing	730(11.66)	12,944(5.55)

### Signal values associated with different immunotherapy regimens

In general, ICI immunotherapies were significantly associated with over-reporting frequencies of endocrine AEs, corresponding to IC_025_ = 2.49 and ROR_025_ = 5.99 (Table 2). When further analysis, higher reporting frequency of endocrine adverse events were observed in all ICI regimens compared with the whole database. For monotherapy, a majority of endocrine complications were reported for anti-PD-1agents (*N* = 3398,54.28%), corresponding to IC_025_ = 2.20 and ROR_025_ = 4.82. By contrary, anti-CTLA-4 drugs contributed a small proportion (*N* = 708, 11.31%) but stronger signal values (IC_025_ = 2.84, ROR_025_ = 7.68), especially ipilimumab holding the strongest signal of ICI-associated endocrine AEs (IC_025_ = 2.84, ROR_025_ = 7.69). A different reporting frequency (i.e., statistically significant ROR) emerged when anti-CTLA-4 therapy were compared with anti-PD-1/PD-L1 treatments for endocrine toxicities, corresponding to ROR = 1.68 (95%CI 1.55–1.83), ROR = 2.54 (95%CI 2.20–2.93), respectively.

**Table 2:** The associations of endocrine AEs with different immunotherapy regimens*

Strategy	Drug	N	IC	IC_025_	IC_975_	ROR	ROR_025_	ROR_975_
Total	Total ICIs	6260	2.53	**2.49**	2.57	6.14	**5.99**	6.30
Monotherapy	Anti-PD-1	3398	2.26	**2.20**	2.31	4.99	**4.82**	5.16
	Nivolumab	2219	2.24	**2.17**	2.31	4.90	**4.70**	5.12
	Pembrolizumab	1175	2.29	**2.19**	2.39	5.07	**4.78**	5.38
	Cemiplimab	4	1.60	−0.31	3.50	3.07	**1.13**	8.32
	Anti-PD-L1	269	1.68	**1.48**	1.88	3.26	**2.89**	3.68
	Atezolizumab	175	1.54	**1.29**	1.79	2.95	**2.54**	3.43
	Avelumab	27	1.66	**1.01**	2.31	3.22	**2.20**	4.73
	Durvalumab	67	2.07	**1.66**	2.47	4.31	**3.37**	5.49
	Anti-CTLA-4	708	2.97	**2.84**	3.09	8.29	**7.68**	8.95
	Ipilimumab	706	2.97	**2.84**	3.09	8.30	**7.69**	8.96
	Tremelimumab	2	1.55	−1.49	4.59	2.98	0.72	12.33
Anti-CTLA-4 vs anti-PD-1		708				1.68	**1.55**	1.83
Anti-CTLA-4 vs anti-PD-L1		708				2.54	**2.20**	2.93
Polytherapy	Polytherapy1	64	4.41	**4.00**	4.83	25.60	**19.44**	33.71
	Polytherapy2	1664	3.15	**3.07**	3.24	9.58	**9.11**	10.07
	Polytherapy3	109	4.05	**3.73**	4.36	18.93	**15.45**	23.20
	Polytherapy4	27	3.96	**3.31**	4.61	17.68	**11.67**	26.78
Polytherapy vs. Monotherapy		1864				2.00	**1.89**	2.11

*In Table 2, bold text denotes significant signals. Polytherapy1: Nivolumab+ pembrolizumab+ ipilimumab; Polytherapy2: Nivolumab+ ipilimumab; Polytherapy3: Pembrolizumab+ ipilimumab; Polytherapy4: Durvalumab+ tremelimumab. N: number of records; IC_025_: the lower end of the 95% confidence interval of IC. IC_975_: the upper end of the 95% confidence interval of IC. ROR_025_: the lower end of the 95% confidence interval of ROR. ROR_975_: the upper end of the 95% confidence interval of IC

For combination therapy, nivolumab+ipilimumab was the most common combination therapy (*N* = 1664,26.58%) also with a strong signal, corresponding to IC_025_ = 3.07, ROR_025_ = 9.11. By contrary, nivolumab+ pembrolizumab+ ipilimumab, despite a very small proportion (*N* = 64,1.02%), presented the strongest signal, corresponding to IC_025_ = 4.00, ROR_025_ = 19.44. Disproportionate reporting was found when comparing combination therapy with monotherapy regimens, in addition, endocrine AEs were over-reported for patients treated with combination therapy versus those treated with monotherapy (ROR = 2.00, 95%CI 1.89–2.11).

### The spectrum of endocrine AEs differs in immunotherapy regimens

**Fig. 1:**
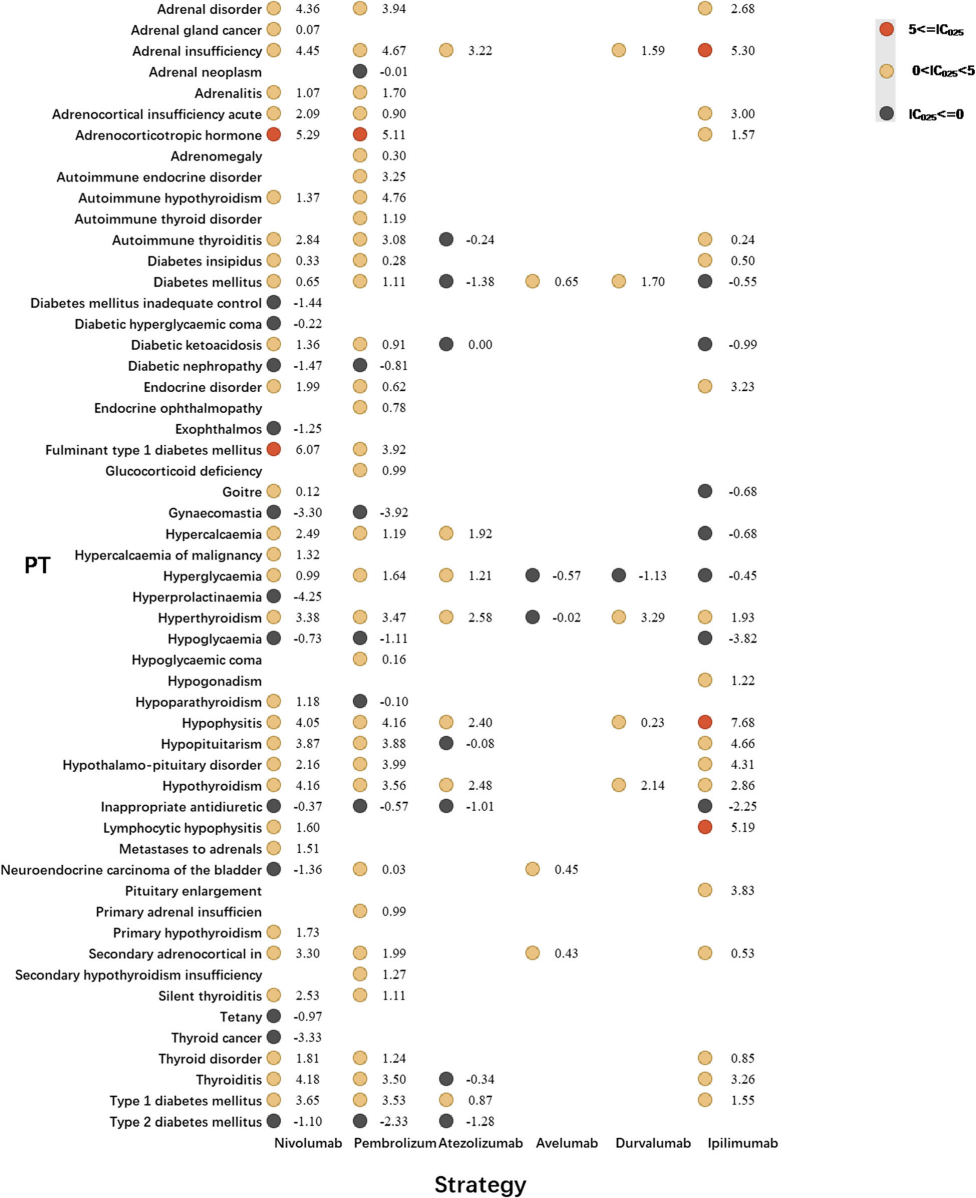
Endocrine toxicity profiles for different ICI monotherapy strategies*. *In Fig. 1, PT: preferred term; IC: information component; IC_025_: the lower end of the 95% confidence interval of IC. IC_025_ greater than 0 was deemed a signal

**Fig. 2:**
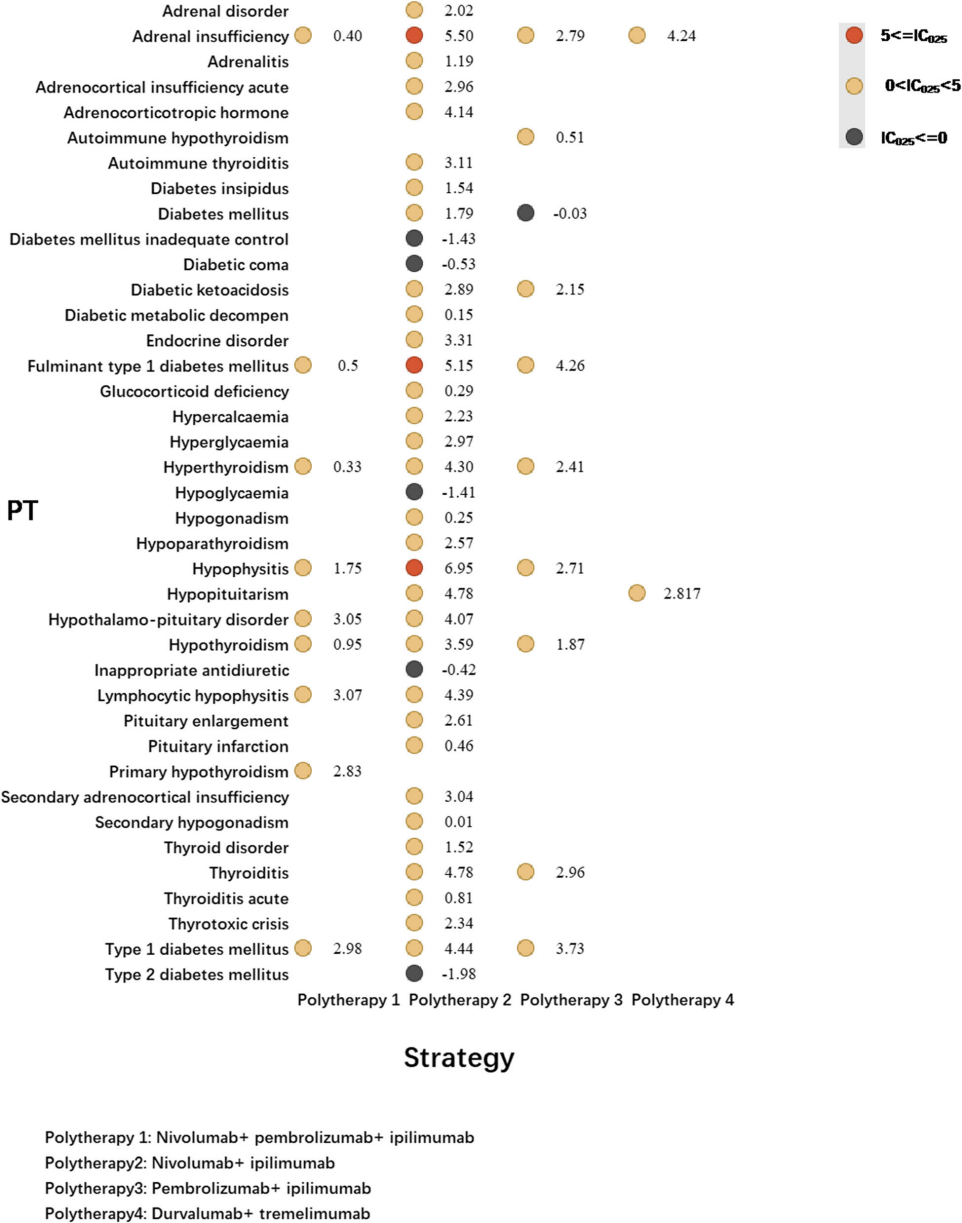
Endocrine toxicity profiles for different ICI combination therapy strategies*. *In Fig. 2, PT: preferred term; IC: information component; IC_025_: the lower end of the 95% confidence interval of IC. IC_025_ greater than 0 was deemed a signal

Tremelimumab has not been approved by FDA and cemiplimab received approval in September 2018 only to treat patients with metastatic or locally advanced cutaneous squamous cell carcinoma who are not candidates for surgery or radiation [12]. Both medications were rarely used, consequently, small number of AEs reported. Therefore, cemiplimab and tremelimumab were not included in further analysis. Figures 1 and 2 presented the endocrine toxicity profiles of different immunotherapy regimens. Full list of endocrine AEs for ICIs can be accessed in additional files (Additional file 1: Table S2 and S3).

Pembrolizumab was with a broadest spectrum of endocrine AEs with 33 PTs detected as signals, ranging from neuroendocrine carcinoma of the bladder (IC_025_ = 0.03) to adrenocorticotropic hormone (IC_025_ = 5.11) (Fig. 1). By contrary, 31 PTs were significantly associated with nivolumab treatment, ranging from adrenal gland cancer (IC_025_ = 0.07) to fulminant type 1 diabetes mellitus (IC_025_ = 6.07). There were 24 PTs both significant associated with pembrolizumab and nivolumab receiving. Among these, most common ones were hypothyroidism, adrenal insufficiency and hyperthyroidism. Endocrine toxicity profiles of anti-PD-L1 drugs varies a lot. Adrenal insufficiency events were found significantly associated with atezolizumab (IC_025_ = 3.22) and durvalumab (IC_025_ = 1.59). Avelumab (IC_025_ = 0.65) and durvalumab (IC_025_ = 1.70) were detected significantly associated with increasing diabetes mellitus events. Regarding anti-CTLA-4 (ipilimumab),19 PTs were observed having significant associations with ipilimumab (overlapping in 15PTs with nivolumab and pembrolizumab). Among these, hypophysitis is most frequent PT, also detected as strongest signal, corresponding to IC_025_ = 7.68. In addition, distinct spectrum of endocrine toxic events also differed markedly between combination therapy regimens (Fig. 2). Nivolumab+ ipilimumab had the widest distribution of endocrine-related irAEs with a total of 32 PTs detected as signals ranging from secondary hypogonadism (IC_025_ = 0.01) to hypophysitis (IC_025_ = 6.95).

**Fig. 3:**
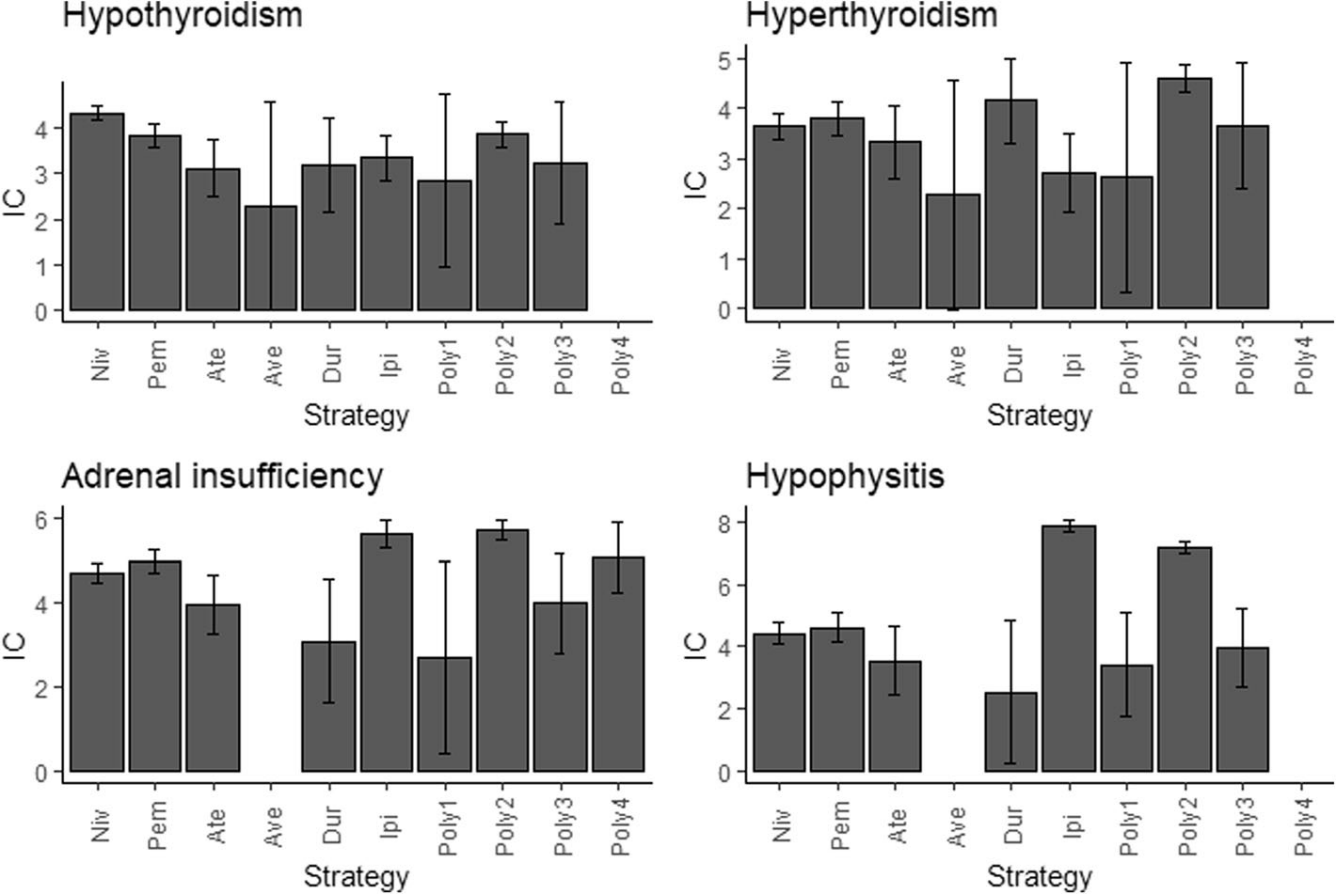
Associations between four top ranked PTs and different ICI strategies quantified by IC value*. *In Fig. 3, PT: preferred term; IC: information component; Niv: nivolumab; Pem: pembrolizumab; Ate: atezolizumab; Ave: avelumab; Dur: durvalumb; Ipi: Ipilimumab; Poly1: Nivolumab+ pembrolizumab+ ipilimumab; Poly2: Nivolumab+ ipilimumab; Poly3: Pembrolizumab+ ipilimumab; Poly4: Durvalumab+ tremelimumab. IC_025_ greater than 0 was deemed a signal

According to our analysis, hypothyroidism (*N* = 885,14.14%), adrenal insufficiency (*N* = 730, 11.66%), hypophysitis (*N* = 688, 10.99%) and hyperthyroidism (*N* = 472, 7.54%) were most common 4 endocrine events after receiving ICI medications (Addition file 1: Table S4) and their correlations with different ICI therapies were also differed. Hypothyroidism and hyperthyroidism seem to be much stronger associated with PD-1 antibodies and nivolumab+ ipilimumab regimen (Fig. 3). Ipilimumab alone or combined nivolumab showing strongest associations with adrenal insufficiency and hypophysitis events. Adrenal insufficiency was the only endocrine complication significantly over-reported in four polytherapy regimens, and it appears to be more strongly associated with nivolumab+ ipilimumab (IC_025_ = 5.50). Notably, patients receiving combination of nivolumab and ipilimumab therapy is highly associated with developing these four ICI-related endocrinopathies.

## Discussion

Monoclonal antibodies (anti-PD-1/anti-PD-L1 and anti-CTLA-4) have brought about a significant breakthrough in the treatment of multiple cancers. Their side effects are equally fascinating as irAEs have been reported in almost all systems [13]. Endocrinopathies are most common irAEs and often irreversible [14]. Prior studies have suggested that about 5–10% of patients treated with ICIs are likely to experience endocrine irAEs of any grade [15]. Nevertheless, the detail risk of experiencing such AEs following the use of ICI regimens are not clearly quantified. To our knowledge, this is the largest and most extensive pharmacovigilance study on endocrine irAEs associated with ICIs leveraging FAERS database. Our study provided more precise data on the endocrine profiles of ICI therapy. In general, there were four main findings observed in our study.

1. Remarkably, we found male accounted for a larger proportion of ICIs-related endocrinopathies than female. It has reported that compared to male, female tend to trigger and sustain a stronger immune response against infections and have an increased propensity to experience autoimmune diseases [16]. Therefore, theoretically, female are more likely to experience ICI-related AEs [17], and consequently might with higher reporting frequencies than male. To explore the effect of gender on the reporting frequencies of endocrine complications after ICIs initiation, we further conducted disproportionality analysis.

In general, male had a slightly lower reporting frequencies compared with female but not significant (ROR = 0.98 95%CI: 0.93–1.04) (Additional file 1: Table S1;Fig. S1). Considering the most common consequences observed in the study, the reporting frequencies also varied. Male have significant lower reporting frequencies in hypothyroidism (ROR = 0.68, 95%CI:0.59–0.78) and hyperthyroidism (ROR = 0.77, 95%CI:0.63–0.93) compared with female, which was consistent with a prior retrospective study demonstrated that thyroid disorders associated with ICIs immunotherapy were more common in female [18]. Regarding hypophysitis, which has been reported at higher rates among male [5], a slightly higher but not significant reporting frequencies was observed in male compared with female (ROR = 1.15, 95%CI:0.96–1.38).

It has been reported among patients with non-small cell lung cancer, males had significantly higher odds of receiving anti-PD1 treatment compared with females [19]. Moreover, both melanoma and non-small cell lung cancer, the two most common reasons that a person would be exposed to ICI therapy, occurring at higher rates in male than in female [5, 20, 21]. Consequently, we tried to explore the gender difference of reporting frequencies in the recipients of ICIs for the therapy of melanoma and non-small cell lung cancer and the results became more complex. For patients receiving ICIs for melanoma and other reasons, the reporting frequencies of endocrinopathies in male and female were comparable, corresponding ROR = 0.91(95%CI:0.84–1.00) and ROR = 1.06(95%CI:0.97–1.15), respectively. By contrast, for individuals receiving ICIs for non-small cell lung cancer, male tended to higher reporting frequencies for endocrine diseases compared with female (ROR = 1.16, 95%CI:1.01–1.33). These results suggesting that sex was a fundamental biological variable and it appeared that gender difference for endocrine irAEs may differ for different cancer/tumor types, as well as specific organs, but the exact factors medicated this observed difference was not clear which deserves more attention in oncology.

Actually, we found studies adequately quantifying the gender difference on ICIs-related irAEs or toxicities were scarce. A systematic review concluded that patients who died of ICIs-associated toxic effects were with similar sex distribution (57% vs 60% male; *χ2* = 0.09; *p* = 0.77) [22]. A few studies have evaluated gender disparity in specific endocrinopathies, and most results were derived from retrospective studies with limited individuals involved. Nonetheless, compared with existing studies, our research based on tremendous records in FAERS may offer some useful clinical evidence and future studies are warranted to monitor and research for these gender difference in the recipient of ICIs.

2. Importantly, our study evaluated and compared the signal strength of endocrine AEs in different immunotherapy regimens. Higher reports of endocrine AEs were observed in all ICI regimens compared with the whole database. It appears that potential endocrine irAEs were more likely to occur in patients in anti-CTLA-4 inhibitors monotherapy group than in anti-PD-1/anti-PD-L1 monotherapy groups. Prior studies [23, 24] have concluded that treatment with anti-PD-1/anti-PD-L1 antibodies therapy appears to result in fewer irAEs than with ipilimumab.

Additionally, another analysis [25] based on FAERS database also support our results suggesting anti-CTLA-4 treatment was associated with a higher reporting frequency of endocrine disorders when compared with anti-PD1/anti-PD-L1 treatment (ROR = 1.60, 95%CI 1.46–1.75). Studies from basic research suggesting that blockade of PD-1 is expected to affect a more restricted repertoire of T cells than that affected by CTLA-4 inhibition [23, 26]. This is likely the reason why immune adverse events seem less frequent with anti-PD-1 or anti-PD-L1 antibodies. Notably, our study revealed combining these agents appears to further increase the risk of ICI-related endocrinopathies. This was concordant to what is observed in prior studies [5, 13], whereas, precise mechanisms underlying these endocrine irAEs remain to be elucidated. Indeed, not only endocrine system, combination therapy was reported to associate with higher toxicity involving multiple organ systems [27]. Therefore, despite combination therapy has shown impressive activity in several common cancers [28–32], it also carried a higher risk of toxicity which should be fully and properly recognized.

3. Notably, our study observed the endocrine adverse event profiles of PD-1, PD-L1 and CTLA-4 targeting medications differed and anti-PD-1 drugs appeared to associate with more endocrine toxicities.

Actually, an adequate comparison between toxicity profiles of anti-PD-1 and anti-PD-L1 agents is difficult [33]. PD-L1 blocking antibodies are much less frequently used than PD-1 blocking antibodies, because these medications are approved later. Additionally, they are also differed in FDA-approved indications and tumor types. A study through meta-analysis and systematic review of the literature confirmed that the incidence of irAEs is higher in patients treated with CTLA-4 medications than in those treated with PD-1 and is lowest in patients receiving PD-L1 drugs [34]. Moreover, in another systematic review, Khoja et al. shown that CTLA-4 and PD-1 monoclonal antibodies have different irAE profiles, which may also differ according to tumor types. They were unable to discriminate the adverse event profiles of the anti-PD-L1 antibodies from those of anti-PD-1 antibodies. However, the authors advanced the hypothesis that anti-PD-L1 antibodies, theoretically, might be less toxic owing to PD-L2 sparing which preserves normal immune homeostasis [35]. needed to be further investigated in future research. Our research might provide some new clues for future research but the exact mechanism behind these observations needed to be further investigated.

4. In addition, our study also provides more precise data on the frequency, spectrum of endocrine irAEs induced by different ICI regimens. Pituitary, thyroid, and adrenal glands are endocrine organs typically affected by ICIs treatment [36]. Accordingly, our analysis demonstrated that hypothyroidism, adrenal insufficiency, hypophysitis and hyperthyroidism were the most frequently occurring endocrine irAEs following the ICIs use. Data from clinical trials focusing on ICIs also present similar results [37]. What’s more, a prior meta-analysis [38] also demonstrated that ICIs are associated with increased risk of these four specific AEs compared with placebo or chemotherapy.

Thyroid dysfunction is one of the most common endocrine-related irAEs associated with ICI treatment, which often presented as hyperthyroidism or hypothyroidism. It is thought to mainly associated with anti-PD-1 therapy as well as combination therapy of anti-PD-1 and anti-CTLA-4 [5]. Evidence from our study also favors this point. Our study demonstrated a higher association of hypothyroidism/ hypothyroidism among patients who received nivolumab or pembrolizumab compared with ipilimumab monotherapy. A prior pharmacovigilance study [39] also showed similar result. A meta-analysis [40] also reported that several types of thyroid dysfunction seem to be more strongly associated with anti-PD-1 treatment or ipilimumab plus nivolumab therapy than ipilimumab alone. Additionally, in our study, hypothyroidism was observed to have a much higher reporting frequency than hyperthyroidism (885 vs 472), and this in accord with results from clinical trials that hypothyroidism occurs more frequently than hyperthyroidism [15].

Adrenal insufficiency is an endocrine disorder usually characterized by the adrenal cortex not producing enough hormone cortisol. ICI-associated adrenal insufficiency can be life-threatening if not early recognized and promptly managed [13]. Our research showed that adrenal insufficiency was with secondary reporting frequency among all endocrine AEs after ICIs. Additionally, a stronger association with adrenal insufficiency emerged for ipilimumab alone or combined with nivolumab. More attention should be focused on it for the potential dehydration, hypotension, and electrolyte imbalances events it may trigger. Individuals on ICI therapy should also be informed about the potential danger of this complication, and prompt diagnosis and treatment are essential.

Hypophysitis is more frequently occurred in patients on anti-CTLA-4 therapy and can affect up to 10% of patients [15]. It is reported that hypophysitis is particularly associated with anti-CTLA-4 therapy [5]. In this study, we noticed that ipilimumab alone as well as combined with nivolumab showing higher risk of developing hypophysitis compared with other regimens and this trend has also been revealed in another study [41]. It is reported that adrenal insufficiency can be triggered by ICI-related hypophysitis [5], which could be life-threating. Thus, special care should be given to individuals (especially on ipilimumab/ ipilimumab+ nivolumab therapy) with this symptom.

Several limitations in our study should also be recognized. Firstly, detail information on clinical data which might contribute to a better comprehensive evaluation of the response rate of the patients associated with these irAEs and durability of the response was missing a lot in FAERS database. Secondly, when a report involves several drugs and/or several adverse events, we took combination of drug–adverse event pair as the basic unit rather than report, so results from this pharmacovigilance analysis may subject bias. Nonetheless, compared with existing studies, strength of enormous records at a national level supports our study quantify the potential risk but truly risk of these events should be ascertained in prospective studies.

## Conclusions

With the increased usage of ICIs recent years, ICI-associated endocrinopathies are on the rise. This study comprehensively evaluated the association of ICIs and potential endocrinopathies from real-world practice. Most of our results were consistent with prior literatures. Clinicians should be aware of the distinct endocrine toxicity profiles of different regimens and patients on ICI medications should be informed of these potential toxicities.

## Supplementary information

**Additional file 1: Table S1.** Reporting frequencies of ICIs-associated (total and common) endocrine events in male compared with female. **Figure S1.** Comparisons of ICIs-related endocrine events (total and common) between male and female. **Table S2.** Disproportionality analysis results for ICI monotherapy strategies and endocrine complications. **Table S3.** Disproportionality analysis results for ICI combination therapy strategies and endocrine complications. **Table S4.** PTs related to endocrine AEs after receiving ICIs in FAERS 2014 Q1-2019Q1, by descending frequency.

## Data Availability

All data is publicly available in website of https://fis.fda.gov/extensions/FPD-QDE-FAERS/FPD-QDE-FAERS.html.

## Abbreviations

`IC: Information component
AEs: Adverse events
FAERS: FDA Adverse Event Reporting System
IC_025_: Lower end of the 95% confidence interval of information component
ICI(s): Immune-checkpoint inhibitor(s)
irAEs: Immune-related adverse events
MedDRA: Medical Dictionary for Regulatory Activities
N: Number of records
PTs: Preferred terms
Q1: First quarter
ROR: Reporting odds ratios
ROR_025_: Lower limit of the 95% confidence interval of reporting odds ratios
