# Supplementary material for: Endocrine toxicity of immune checkpoint inhibitors: a real-world study leveraging US Food and Drug Administration adverse events reporting system
Source: J Immunother Cancer. 2019 Nov 6;7:286. doi: 10.1186/s40425-019-0754-2 (PMC6836403; doi:10.1186/s40425-019-0754-2)
Supplement: Supplementary file 1 — Additional file 1: Table S1. Reporting frequencies of ICIs-associated (total and common) endocrine events in male compared with female. Figure S1. Comparisons of ICIs-related endocrine events (total and common) between male and female. Table S2. Disproportionality analysis results for ICI monotherapy strategies and endocrine complications. Table S3. Disproportionality analysis results for ICI combination therapy strategies and endocrine complications. Table S4. PTs related to endocrine AEs after receiving ICIs in FAERS 2014 Q1-2019Q1, by descending frequency. [file 40425_2019_754_MOESM1_ESM.docx]

**Table S1** Reporting frequencies of ICIs-associated (total and common) endocrine events in male compared with female*

|  | **a** | **b** | **c** | **d** | **ROR** | **ROR_025_** | **ROR_975_** |
| --- | --- | --- | --- | --- | --- | --- | --- |
| **Total**  **(N=29294335)** | 3428 | 69180 | 2095 | 41550 | 0.98 | 0.93 | 1.04 |
| Hypothyroidism | 425 | 72183 | 374 | 43271 | 0.68 | 0.59 | 0.78 |
| Adrenal insufficiency | 447 | 72161 | 203 | 43442 | 1.33 | 1.12 | 1.56 |
| Hypophysitis | 336 | 72272 | 176 | 43469 | 1.15 | 0.96 | 1.38 |
| Hyperthyroidism | 241 | 72367 | 189 | 43456 | 0.77 | 0.63 | 0.93 |
| **Melanoma**  **(N=136963)** | 1235 | 15238 | 950 | 10694 | 0.91 | 0.84 | 1.00 |
| Hypothyroidism | 124 | 16349 | 169 | 11475 | 0.52 | 0.41 | 0.65 |
| Adrenal Insufficiency | 138 | 16335 | 79 | 11565 | 1.24 | 0.94 | 1.63 |
| Hypophysitis | 210 | 16263 | 101 | 11543 | 1.47 | 1.16 | 1.87 |
| Hyperthyroidism | 79 | 16394 | 81 | 11563 | 0.69 | 0.51 | 0.94 |
| **Non-small cell lung cancer (N=190307)** | 706 | 14710 | 296 | 7129 | 1.16 | 1.01 | 1.33 |
| Hypothyroidism | 108 | 15308 | 52 | 7373 | 1.00 | 0.72 | 1.39 |
| Adrenal Insufficiency | 102 | 15314 | 32 | 7393 | 1.53 | 1.03 | 2.29 |
| Hypophysitis | 33 | 15383 | 13 | 7412 | 1.22 | 0.64 | 2.32 |
| Hyperthyroidism | 63 | 15353 | 33 | 7392 | 0.92 | 0.60 | 1.40 |
| **Other indications**  **(N=28967065)** | 1487 | 39232 | 849 | 23727 | 1.06 | 0.97 | 1.15 |
| Hypothyroidism | 193 | 40526 | 153 | 24423 | 0.76 | 0.61 | 0.94 |
| Adrenal Insufficiency | 207 | 40512 | 92 | 24484 | 1.36 | 1.06 | 1.74 |
| Hypophysitis | 93 | 40626 | 62 | 24514 | 0.91 | 0.66 | 1.25 |
| Hyperthyroidism | 99 | 40620 | 75 | 24501 | 0.80 | 0.59 | 1.08 |

* In **Table S1**, N: number of total records; a: the number of records with endocrine AEs reported for ICIs; b: the number of records with any other AEs reported for ICIs; c: the number of records with any endocrine AEs for other drugs; d: the number of records reported other AEs for other drugs. ROR_025_: the lower end of the 95% confidence interval of ROR. ROR_975_: the upper end of the 95% confidence interval of ROR. ROR_025_ exceeds 1 was deemed significant difference.

**
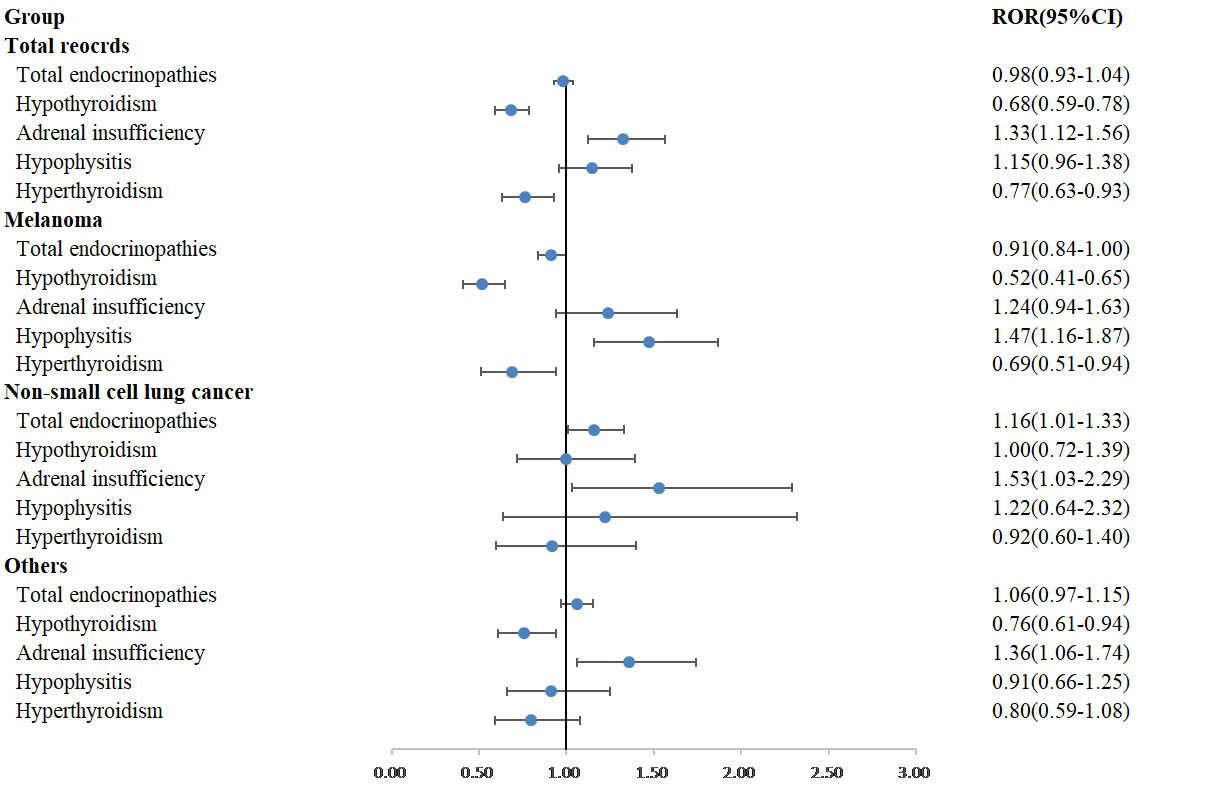
**

**Figure S1** Comparisons of ICIs-related endocrine events (total and common) between male and female.

**Table S2** Disproportionality analysis results for ICI monotherapy strategies and endocrine complications*

| **Drug** | **PT** | **a** | **b** | **c** | **d** | **IC** | **IC_025_** | **IC_975_** | **ROR** | **ROR_025_** | **ROR_975_** |
| --- | --- | --- | --- | --- | --- | --- | --- | --- | --- | --- | --- |
| Nivolumab | Hypothyroidism | 466 | 56997 | 11260 | 29225613 | 4.311 | **4.158** | 4.464 | 20.771 | **18.926** | 22.795 |
|  | Adrenal insufficiency | 222 | 57241 | 3965 | 29232908 | 4.674 | **4.452** | 4.897 | 26.924 | **23.514** | 30.829 |
|  | Hyperthyroidism | 167 | 57296 | 6434 | 29230439 | 3.639 | **3.382** | 3.895 | 12.775 | **10.953** | 14.900 |
|  | Fulminant type 1 diabetes mellitus | 130 | 57333 | 425 | 29236448 | 6.360 | **6.069** | 6.651 | 97.868 | **80.399** | 119.132 |
|  | Diabetic ketoacidosis | 111 | 57352 | 17452 | 29219421 | 1.674 | **1.359** | 1.989 | 3.208 | **2.662** | 3.867 |
|  | Diabetes mellitus | 110 | 57353 | 28504 | 29208369 | 0.964 | **0.648** | 1.281 | 1.957 | **1.622** | 2.360 |
|  | Type 1 diabetes mellitus | 103 | 57360 | 2988 | 29233885 | 3.979 | **3.652** | 4.306 | 16.266 | **13.363** | 19.800 |
|  | Hypercalcaemia | 89 | 57374 | 6037 | 29230836 | 2.838 | **2.486** | 3.190 | 7.247 | **5.878** | 8.936 |
|  | Thyroiditis | 85 | 57378 | 1528 | 29235345 | 4.544 | **4.184** | 4.905 | 24.436 | **19.639** | 30.406 |
|  | Hyperglycaemia | 83 | 57380 | 16287 | 29220586 | 1.356 | **0.992** | 1.721 | 2.571 | **2.072** | 3.190 |
|  | Hypophysitis | 81 | 57382 | 1602 | 29235271 | 4.422 | **4.053** | 4.792 | 22.363 | **17.887** | 27.960 |
|  | Thyroid disorder | 73 | 57390 | 7826 | 29229047 | 2.200 | **1.811** | 2.590 | 4.633 | **3.678** | 5.834 |
|  | Adrenal disorder | 52 | 57411 | 637 | 29236236 | 4.826 | **4.363** | 5.288 | 29.985 | **22.599** | 39.785 |
|  | Adrenocorticotropic hormone deficiency | 52 | 57411 | 188 | 29236685 | 5.757 | **5.294** | 6.220 | 60.403 | **44.426** | 82.125 |
|  | Hypopituitarism | 50 | 57413 | 967 | 29235906 | 4.339 | **3.867** | 4.812 | 21.051 | **15.840** | 27.975 |
|  | Hypoglycaemia | 43 | 57420 | 25532 | 29211341 | -0.220 | -0.730 | 0.290 | 0.858 | 0.636 | 1.158 |
|  | Autoimmune thyroiditis | 34 | 57429 | 1358 | 29235515 | 3.417 | **2.841** | 3.993 | 10.892 | **7.749** | 15.308 |
|  | Secondary adrenocortical insufficiency | 33 | 57430 | 866 | 29236007 | 3.888 | **3.303** | 4.473 | 15.219 | **10.749** | 21.548 |
|  | Type 2 diabetes mellitus | 29 | 57434 | 20594 | 29216279 | -0.473 | -1.099 | 0.152 | 0.720 | 0.500 | 1.036 |
|  | Inappropriate antidiuretic hormone secretion | 20 | 57443 | 7702 | 29229171 | 0.390 | -0.371 | 1.150 | 1.311 | 0.845 | 2.033 |
|  | Adrenocortical insufficiency acute | 15 | 57448 | 731 | 29236142 | 2.981 | **2.094** | 3.867 | 8.005 | **4.800** | 13.347 |
|  | Hypothalamo-pituitary disorder | 15 | 57448 | 686 | 29236187 | 3.047 | **2.161** | 3.934 | 8.388 | **5.028** | 13.991 |
|  | Endocrine disorder | 13 | 57450 | 625 | 29236248 | 2.946 | **1.988** | 3.904 | 7.812 | **4.510** | 13.530 |
|  | Gynaecomastia | 13 | 57450 | 34690 | 29202183 | -2.345 | -3.303 | -1.387 | 0.196 | 0.114 | 0.338 |
|  | Diabetes mellitus inadequate control | 11 | 57452 | 7417 | 29229456 | -0.390 | -1.440 | 0.660 | 0.763 | 0.422 | 1.378 |
|  | Goitre | 9 | 57454 | 1708 | 29235165 | 1.296 | **0.122** | 2.470 | 2.463 | **1.279** | 4.743 |
|  | Silent thyroiditis | 8 | 57455 | 52 | 29236821 | 3.782 | **2.527** | 5.038 | 14.115 | **6.705** | 29.715 |
|  | Diabetes insipidus | 7 | 57456 | 928 | 29235945 | 1.684 | **0.329** | 3.039 | 3.228 | **1.534** | 6.789 |
|  | Lymphocytic hypophysitis | 7 | 57456 | 231 | 29236642 | 2.956 | **1.600** | 4.311 | 7.862 | **3.706** | 16.677 |
|  | Metastases to adrenals | 7 | 57456 | 262 | 29236611 | 2.868 | **1.512** | 4.223 | 7.390 | **3.488** | 15.655 |
|  | Hypoparathyroidism | 6 | 57457 | 263 | 29236610 | 2.661 | **1.178** | 4.144 | 6.392 | **2.846** | 14.359 |
|  | Primary hypothyroidism | 6 | 57457 | 96 | 29236777 | 3.215 | **1.732** | 4.698 | 9.439 | **4.137** | 21.534 |
|  | Autoimmune hypothyroidism | 5 | 57458 | 85 | 29236788 | 3.023 | **1.368** | 4.678 | 8.245 | **3.346** | 20.320 |
|  | Hypercalcaemia of malignancy | 5 | 57458 | 98 | 29236775 | 2.970 | **1.315** | 4.624 | 7.941 | **3.233** | 19.506 |
|  | Adrenalitis | 4 | 57459 | 33 | 29236840 | 2.974 | **1.074** | 4.874 | 7.967 | **2.822** | 22.488 |
|  | Tetany | 4 | 57459 | 949 | 29235924 | 0.925 | -0.975 | 2.825 | 1.903 | 0.713 | 5.080 |
|  | Adrenal gland cancer | 3 | 57460 | 90 | 29236783 | 2.359 | **0.068** | 4.649 | 5.171 | **1.637** | 16.336 |
|  | Diabetic hyperglycaemic coma | 3 | 57460 | 167 | 29236706 | 2.070 | -0.220 | 4.360 | 4.226 | **1.349** | 13.237 |
|  | Diabetic nephropathy | 3 | 57460 | 754 | 29236119 | 0.818 | -1.472 | 3.108 | 1.766 | 0.568 | 5.488 |
|  | Exophthalmos | 3 | 57460 | 607 | 29236266 | 1.045 | -1.245 | 3.335 | 2.067 | 0.665 | 6.428 |
|  | Hyperprolactinaemia | 3 | 57460 | 6667 | 29230206 | -1.956 | -4.247 | 0.334 | 0.257 | 0.083 | 0.798 |
|  | Neuroendocrine carcinoma of the bladder | 3 | 57460 | 676 | 29236197 | 0.934 | -1.356 | 3.224 | 1.914 | 0.616 | 5.950 |
|  | Thyroid cancer | 3 | 57460 | 3423 | 29233450 | -1.045 | -3.335 | 1.245 | 0.484 | 0.156 | 1.502 |
| Pembrolizumab | Hypothyroidism | 172 | 29158 | 11554 | 29253452 | 3.817 | **3.564** | 4.069 | 14.356 | **12.344** | 16.695 |
|  | Adrenal insufficiency | 144 | 29186 | 4043 | 29260963 | 4.945 | **4.669** | 5.221 | 31.880 | **26.987** | 37.660 |
|  | Hyperthyroidism | 99 | 29231 | 6502 | 29258504 | 3.807 | **3.473** | 4.141 | 14.223 | **11.658** | 17.351 |
|  | Diabetes mellitus | 81 | 29249 | 28533 | 29236473 | 1.483 | **1.114** | 1.853 | 2.806 | **2.255** | 3.491 |
|  | Hyperglycaemia | 69 | 29261 | 16301 | 29248705 | 2.041 | **1.640** | 2.442 | 4.135 | **3.263** | 5.239 |
|  | Type 1 diabetes mellitus | 56 | 29274 | 3035 | 29261971 | 3.974 | **3.529** | 4.420 | 15.977 | **12.263** | 20.816 |
|  | Hypophysitis | 53 | 29277 | 1630 | 29263376 | 4.614 | **4.155** | 5.072 | 25.108 | **19.094** | 33.017 |
|  | Diabetic ketoacidosis | 47 | 29283 | 17516 | 29247490 | 1.393 | **0.906** | 1.881 | 2.633 | **1.977** | 3.507 |
|  | Adrenocorticotropic hormone deficiency | 37 | 29293 | 203 | 29264803 | 5.663 | **5.111** | 6.214 | 53.328 | **37.559** | 75.717 |
|  | Thyroiditis | 35 | 29295 | 1578 | 29263428 | 4.069 | **3.502** | 4.637 | 17.070 | **12.209** | 23.866 |
|  | Hypopituitarism | 33 | 29297 | 984 | 29264022 | 4.464 | **3.879** | 5.049 | 22.557 | **15.943** | 31.916 |
|  | Thyroid disorder | 30 | 29300 | 7869 | 29257137 | 1.859 | **1.244** | 2.474 | 3.639 | **2.542** | 5.210 |
|  | Hypothalamo-pituitary disorder | 29 | 29301 | 672 | 29264334 | 4.617 | **3.992** | 5.243 | 25.153 | **17.341** | 36.484 |
|  | Adrenal disorder | 28 | 29302 | 661 | 29264345 | 4.582 | **3.945** | 5.219 | 24.530 | **16.803** | 35.810 |
|  | Autoimmune hypothyroidism | 25 | 29305 | 65 | 29264941 | 5.433 | **4.757** | 6.109 | 45.126 | **28.447** | 71.583 |
|  | Autoimmune thyroiditis | 25 | 29305 | 1367 | 29263639 | 3.751 | **3.075** | 4.427 | 13.644 | **9.185** | 20.268 |
|  | Fulminant type 1 diabetes mellitus | 25 | 29305 | 530 | 29264476 | 4.594 | **3.918** | 5.270 | 24.740 | **16.562** | 36.955 |
|  | Hypercalcaemia | 24 | 29306 | 6102 | 29258904 | 1.885 | **1.194** | 2.576 | 3.705 | **2.481** | 5.534 |
|  | Hypoglycaemia | 20 | 29310 | 25555 | 29239451 | -0.349 | -1.109 | 0.411 | 0.785 | 0.506 | 1.217 |
|  | Inappropriate antidiuretic hormone secretion | 11 | 29319 | 7711 | 29257295 | 0.482 | -0.567 | 1.532 | 1.398 | 0.774 | 2.525 |
|  | Secondary adrenocortical insufficiency | 11 | 29319 | 888 | 29264118 | 3.038 | **1.988** | 4.088 | 8.275 | **4.566** | 14.999 |
|  | Autoimmune endocrine disorder | 10 | 29320 | 3 | 29265003 | 4.355 | **3.248** | 5.462 | 20.875 | **5.745** | 75.854 |
|  | Type 2 diabetes mellitus | 9 | 29321 | 20614 | 29244392 | -1.155 | -2.329 | 0.020 | 0.449 | 0.233 | 0.863 |
|  | Hypoglycaemic coma | 7 | 29323 | 2112 | 29262894 | 1.516 | **0.161** | 2.872 | 2.867 | **1.365** | 6.021 |
|  | Adrenocortical insufficienc | 6 | 29324 | 740 | 29264266 | 2.382 | **0.899** | 3.865 | 5.236 | **2.344** | 11.693 |
|  | Gynaecomastia | 6 | 29324 | 34697 | 29230309 | -2.439 | -3.922 | -0.956 | 0.184 | 0.083 | 0.410 |
|  | Adrenalitis | 5 | 29325 | 32 | 29264974 | 3.356 | **1.702** | 5.011 | 10.337 | **4.027** | 26.532 |
|  | Diabetes insipidus | 5 | 29325 | 930 | 29264076 | 1.937 | **0.283** | 3.592 | 3.841 | **1.595** | 9.251 |
|  | Endocrine disorder | 5 | 29325 | 633 | 29264373 | 2.272 | **0.617** | 3.927 | 4.849 | **2.011** | 11.691 |
|  | Secondary hypothyroidism | 5 | 29325 | 217 | 29264789 | 2.929 | **1.274** | 4.584 | 7.666 | **3.159** | 18.606 |
|  | Autoimmune thyroid disorder | 4 | 29326 | 25 | 29264981 | 3.088 | **1.188** | 4.988 | 8.571 | **2.983** | 24.628 |
|  | Endocrine ophthalmopathy | 4 | 29326 | 198 | 29264808 | 2.680 | **0.780** | 4.580 | 6.443 | **2.394** | 17.339 |
|  | Glucocorticoid deficiency | 4 | 29326 | 104 | 29264902 | 2.887 | **0.987** | 4.787 | 7.448 | **2.743** | 20.219 |
|  | Neuroendocrine carcinoma of the bladder | 4 | 29326 | 675 | 29264331 | 1.931 | **0.031** | 3.831 | 3.825 | **1.431** | 10.222 |
|  | Primary adrenal Primary adrenal insufficiency | 4 | 29326 | 105 | 29264901 | 2.885 | **0.985** | 4.785 | 7.435 | **2.739** | 20.182 |
|  | Silent thyroiditis | 4 | 29326 | 56 | 29264950 | 3.006 | **1.106** | 4.906 | 8.092 | **2.934** | 22.316 |
|  | Adrenal neoplasm | 3 | 29327 | 217 | 29264789 | 2.281 | -0.009 | 4.571 | 4.878 | **1.561** | 15.245 |
|  | Adrenomegaly | 3 | 29327 | 77 | 29264929 | 2.593 | **0.303** | 4.883 | 6.064 | **1.913** | 19.219 |
|  | Diabetic nephropathy | 3 | 29327 | 754 | 29264252 | 1.476 | -0.814 | 3.766 | 2.787 | 0.897 | 8.663 |
|  | Hypoparathyroidism | 3 | 29327 | 266 | 29264740 | 2.186 | -0.104 | 4.476 | 4.566 | **1.463** | 14.248 |
| Atezolizumab | Hypothyroidism | 29 | 7294 | 11697 | 29275316 | 3.104 | **2.478** | 3.730 | 8.640 | **5.997** | 12.448 |
|  | Adrenal insufficiency | 23 | 7300 | 4164 | 29282849 | 3.925 | **3.219** | 4.632 | 15.279 | **10.135** | 23.033 |
|  | Hyperthyroidism | 21 | 7302 | 6580 | 29280433 | 3.322 | **2.581** | 4.063 | 10.042 | **6.539** | 15.422 |
|  | Hyperglycaemia | 18 | 7305 | 16352 | 29270661 | 2.010 | **1.206** | 2.814 | 4.038 | **2.542** | 6.415 |
|  | Hypercalcaemia | 14 | 7309 | 6112 | 29280901 | 2.836 | **1.915** | 3.756 | 7.158 | **4.235** | 12.100 |
|  | Diabetic ketoacidosis | 10 | 7313 | 17553 | 29269460 | 1.102 | -0.004 | 2.209 | 2.149 | **1.156** | 3.997 |
|  | Hypophysitis | 10 | 7313 | 1673 | 29285340 | 3.511 | **2.405** | 4.618 | 11.441 | **6.142** | 21.312 |
|  | Diabetes mellitus | 7 | 7316 | 28607 | 29258406 | -0.029 | -1.384 | 1.326 | 0.980 | 0.467 | 2.057 |
|  | Type 1 diabetes mellitus | 6 | 7317 | 3085 | 29283928 | 2.353 | **0.869** | 3.836 | 5.115 | **2.295** | 11.398 |
|  | Type 2 diabetes mellitus | 6 | 7317 | 20617 | 29266396 | 0.201 | -1.282 | 1.684 | 1.150 | 0.516 | 2.560 |
|  | Inappropriate antidiuretic hormone secretion | 4 | 7319 | 7718 | 29279295 | 0.889 | -1.011 | 2.789 | 1.852 | 0.695 | 4.938 |
|  | Autoimmune thyroiditis | 3 | 7320 | 1389 | 29285624 | 2.045 | -0.245 | 4.335 | 4.131 | **1.330** | 12.828 |
|  | Hypopituitarism | 3 | 7320 | 1014 | 29285999 | 2.214 | -0.076 | 4.504 | 4.645 | **1.495** | 14.431 |
|  | Thyroiditis | 3 | 7320 | 1610 | 29285403 | 1.954 | -0.336 | 4.244 | 3.878 | **1.249** | 12.041 |
| Avelumab | Diabetes mellitus | 6 | 995 | 28608 | 29264727 | 2.137 | **0.654** | 3.620 | 4.414 | **1.978** | 9.849 |
|  | Hyperglycaemia | 3 | 998 | 16367 | 29276968 | 1.724 | -0.566 | 4.014 | 3.308 | **1.065** | 10.277 |
|  | Hyperthyroidism | 3 | 998 | 6598 | 29286737 | 2.270 | -0.020 | 4.560 | 4.829 | **1.554** | 15.001 |
|  | Neuroendocrine carcinoma of the bladder | 3 | 998 | 676 | 29292659 | 2.742 | **0.452** | 5.032 | 6.692 | **2.149** | 20.836 |
|  | Secondary adrenocortical insufficiency | 3 | 998 | 896 | 29292439 | 2.721 | **0.431** | 5.011 | 6.597 | **2.120** | 20.529 |
| Durvalumab | Hyperthyroidism | 16 | 1892 | 6585 | 29285843 | 4.149 | **3.293** | 5.005 | 17.830 | **10.894** | 29.181 |
|  | Diabetes mellitus | 14 | 1894 | 28600 | 29263828 | 2.617 | **1.697** | 3.537 | 6.167 | **3.645** | 10.435 |
|  | Hypothyroidism | 11 | 1897 | 11715 | 29280713 | 3.186 | **2.136** | 4.236 | 9.134 | **5.049** | 16.527 |
|  | Adrenal insufficiency | 6 | 1902 | 4181 | 29288247 | 3.072 | **1.589** | 4.556 | 8.425 | **3.778** | 18.788 |
|  | Hyperglycaemia | 3 | 1905 | 16367 | 29276061 | 1.160 | -1.130 | 3.450 | 2.236 | 0.721 | 6.941 |
|  | Hypophysitis | 3 | 1905 | 1680 | 29290748 | 2.521 | **0.231** | 4.812 | 5.745 | **1.849** | 17.846 |
| Ipilimumab | Hypophysitis | 267 | 10725 | 1416 | 29281928 | 7.885 | **7.683** | 8.088 | 262.607 | **230.102** | 299.703 |
|  | Adrenal insufficiency | 102 | 10890 | 4085 | 29279259 | 5.629 | **5.300** | 5.958 | 50.759 | **41.667** | 61.834 |
|  | Hypothyroidism | 49 | 10943 | 11677 | 29271667 | 3.337 | **2.859** | 3.814 | 10.174 | **7.680** | 13.478 |
|  | Hypopituitarism | 33 | 10959 | 984 | 29282360 | 5.248 | **4.663** | 5.833 | 38.583 | **27.260** | 54.608 |
|  | Lymphocytic hypophysitis | 32 | 10960 | 206 | 29283138 | 5.785 | **5.191** | 6.380 | 56.316 | **38.787** | 81.767 |
|  | Hypothalamo-pituitary disorder | 24 | 10968 | 677 | 29282667 | 5.005 | **4.314** | 5.696 | 32.512 | **21.629** | 48.869 |
|  | Hyperthyroidism | 19 | 10973 | 6582 | 29276762 | 2.712 | **1.930** | 3.493 | 6.572 | **4.188** | 10.315 |
|  | Thyroiditis | 18 | 10974 | 1595 | 29281749 | 4.065 | **3.261** | 4.869 | 16.852 | **10.586** | 26.828 |
|  | Diabetes mellitus | 14 | 10978 | 28600 | 29254744 | 0.368 | -0.552 | 1.288 | 1.291 | 0.764 | 2.181 |
|  | Pituitary enlargement | 14 | 10978 | 93 | 29283251 | 4.747 | **3.826** | 5.667 | 27.110 | **15.451** | 47.564 |
|  | Endocrine disorder | 13 | 10979 | 625 | 29282719 | 4.190 | **3.233** | 5.148 | 18.384 | **10.611** | 31.850 |
|  | Adrenocortical insufficiency acute | 12 | 10980 | 734 | 29282610 | 4.002 | **3.002** | 5.003 | 16.124 | **9.112** | 28.533 |
|  | Thyroid disorder | 12 | 10980 | 7887 | 29275457 | 1.851 | **0.851** | 2.852 | 3.615 | **2.051** | 6.370 |
|  | Adrenal disorder | 10 | 10982 | 679 | 29282665 | 3.791 | **2.684** | 4.898 | 13.914 | **7.450** | 25.985 |
|  | Hyperglycaemia | 10 | 10982 | 16360 | 29266984 | 0.661 | -0.446 | 1.767 | 1.582 | 0.851 | 2.941 |
|  | Type 1 diabetes mellitus | 10 | 10982 | 3081 | 29280263 | 2.661 | **1.554** | 3.768 | 6.342 | **3.408** | 11.803 |
|  | Diabetic ketoacidosis | 8 | 10984 | 17555 | 29265789 | 0.262 | -0.993 | 1.517 | 1.199 | 0.599 | 2.399 |
|  | Adrenocorticotropic hormone | 5 | 10987 | 235 | 29283109 | 3.221 | **1.566** | 4.875 | 9.351 | **3.855** | 22.680 |
|  | Hypercalcaemia | 5 | 10987 | 6121 | 29277223 | 0.975 | -0.680 | 2.629 | 1.966 | 0.818 | 4.727 |
|  | Hypogonadism | 5 | 10987 | 659 | 29282685 | 2.876 | **1.221** | 4.531 | 7.360 | **3.053** | 17.746 |
|  | Autoimmune thyroiditis | 4 | 10988 | 1388 | 29281956 | 2.138 | **0.238** | 4.038 | 4.408 | **1.652** | 11.764 |
|  | Diabetes insipidus | 4 | 10988 | 931 | 29282413 | 2.403 | **0.503** | 4.303 | 5.298 | **1.984** | 14.149 |
|  | Secondary adrenocortical insufficiency | 4 | 10988 | 895 | 29282449 | 2.426 | **0.526** | 4.326 | 5.384 | **2.016** | 14.379 |
|  | Goitre | 3 | 10989 | 1714 | 29281630 | 1.613 | -0.677 | 3.903 | 3.061 | 0.986 | 9.503 |
|  | Hypoglycaemia | 3 | 10989 | 25572 | 29257772 | -1.528 | -3.819 | 0.762 | 0.346 | 0.112 | 1.074 |
|  | Inappropriate antidiuretic hormone secretion | 3 | 10989 | 7719 | 29275625 | 0.043 | -2.247 | 2.333 | 1.030 | 0.332 | 3.195 |

*In **Table S2**, PT: preferred term; a: the number of records with endocrine AEs reported for ICIs; b: the number of records with any other AEs reported for ICIs; c: the number of records with any endocrine AEs for other drugs; d: the number of records reported other AEs for other drugs. IC_025_: the lower end of the 95% confidence interval of IC. IC_975_: the upper end of the 95% confidence interval of IC. IC_025_ greater than 0 was deemed a signal. ROR_025_: the lower end of the 95% confidence interval of ROR. ROR_975_: the upper end of the 95% confidence interval of ROR.ROR_025_ exceeds 1 was deemed a signal. Bold text denotes a significant signal. Only PTs with at least 3 records were listed.

**Table S3** Disproportionality analysis results for ICI combination therapy strategies and endocrine complications*

| **Drug** | **PT** | **a** | **b** | **c** | **d** | **IC** | **IC_025_** | **IC_975_** | **ROR** | **ROR_025_** | **ROR_975_** |
| --- | --- | --- | --- | --- | --- | --- | --- | --- | --- | --- | --- |
| Nivolumab+ pembrolizumab+ ipilimumab | Hypothalamo-pituitary disorder | 9 | 300 | 692 | 29293335 | 4.227 | **3.053** | 5.401 | 18.734 | **9.613** | 36.510 |
|  | Lymphocytic hypophysitis | 9 | 300 | 229 | 29293798 | 4.241 | **3.067** | 5.415 | 18.911 | **9.623** | 37.164 |
|  | Type 1 diabetes mellitus | 9 | 300 | 3082 | 29290945 | 4.157 | **2.983** | 5.331 | 17.872 | **9.200** | 34.717 |
|  | Primary hypothyroidism | 8 | 301 | 94 | 29293933 | 4.084 | **2.829** | 5.339 | 16.967 | **8.171** | 35.231 |
|  | Hypophysitis | 5 | 304 | 1678 | 29292349 | 3.409 | **1.754** | 5.064 | 10.630 | **4.387** | 25.756 |
|  | Hypothyroidism | 4 | 305 | 11722 | 29282305 | 2.851 | **0.951** | 4.751 | 7.234 | **2.697** | 19.401 |
|  | Adrenal insufficiency | 3 | 306 | 4184 | 29289843 | 2.685 | **0.395** | 4.975 | 6.437 | **2.064** | 20.078 |
|  | Fulminant type 1 diabetes mellitus | 3 | 306 | 552 | 29293475 | 2.791 | **0.500** | 5.081 | 6.920 | **2.213** | 21.642 |
|  | Hyperthyroidism | 3 | 306 | 6598 | 29287429 | 2.619 | **0.329** | 4.909 | 6.152 | **1.973** | 19.185 |
| Nivolumab+ ipilimumab | Hypophysitis | 258 | 22543 | 1425 | 29270110 | 7.158 | **6.952** | 7.364 | 161.816 | **141.628** | 184.881 |
|  | Adrenal insufficiency | 199 | 22602 | 3988 | 29267547 | 5.730 | **5.495** | 5.965 | 55.730 | **48.306** | 64.295 |
|  | Hypothyroidism | 140 | 22661 | 11586 | 29259949 | 3.867 | **3.587** | 4.147 | 14.832 | **12.549** | 17.530 |
|  | Hyperthyroidism | 135 | 22666 | 6466 | 29265069 | 4.587 | **4.302** | 4.872 | 24.601 | **20.735** | 29.187 |
|  | Diabetic ketoacidosis | 128 | 22673 | 17435 | 29254100 | 3.181 | **2.888** | 3.474 | 9.170 | **7.703** | 10.917 |
|  | Hyperglycaemia | 127 | 22674 | 16243 | 29255292 | 3.267 | **2.973** | 3.562 | 9.741 | **8.176** | 11.605 |
|  | Diabetes mellitus | 99 | 22702 | 28515 | 29243020 | 2.127 | **1.794** | 2.461 | 4.395 | **3.607** | 5.357 |
|  | Type 1 diabetes mellitus | 81 | 22720 | 3010 | 29268525 | 4.810 | **4.440** | 5.179 | 28.732 | **23.033** | 35.841 |
|  | Thyroiditis | 64 | 22737 | 1549 | 29269986 | 5.199 | **4.783** | 5.616 | 37.868 | **29.482** | 48.641 |
|  | Hypopituitarism | 49 | 22752 | 968 | 29270567 | 5.260 | **4.783** | 5.737 | 39.523 | **29.654** | 52.677 |
|  | Fulminant type 1 diabetes mellitus | 46 | 22755 | 509 | 29271026 | 5.641 | **5.148** | 6.134 | 51.915 | **38.381** | 70.221 |
|  | Hypercalcaemia | 36 | 22765 | 6090 | 29265445 | 2.793 | **2.233** | 3.352 | 6.969 | **5.021** | 9.674 |
|  | Thyroid disorder | 29 | 22772 | 7870 | 29263665 | 2.150 | **1.524** | 2.775 | 4.453 | **3.092** | 6.414 |
|  | Hypothalamo-pituitary disorder | 27 | 22774 | 674 | 29270861 | 4.717 | **4.068** | 5.366 | 26.845 | **18.268** | 39.448 |
|  | Lymphocytic hypophysitis | 23 | 22778 | 215 | 29271320 | 5.100 | **4.394** | 5.806 | 35.216 | **22.904** | 54.147 |
|  | Autoimmune thyroiditis | 22 | 22779 | 1370 | 29270165 | 3.829 | **3.106** | 4.552 | 14.366 | **9.426** | 21.896 |
|  | Adrenocorticotropic hormone deficiency | 20 | 22781 | 220 | 29271315 | 4.900 | **4.139** | 5.660 | 30.541 | **19.320** | 48.280 |
|  | Endocrine disorder | 17 | 22784 | 621 | 29270914 | 4.134 | **3.305** | 4.963 | 17.796 | **10.990** | 28.817 |
|  | Secondary adrenocortical insufficiency | 17 | 22784 | 882 | 29270653 | 3.867 | **3.038** | 4.695 | 14.749 | **9.125** | 23.838 |
|  | Adrenocortical insufficiency acute | 15 | 22786 | 731 | 29270804 | 3.842 | **2.956** | 4.729 | 14.499 | **8.694** | 24.179 |
|  | Hypoglycaemia | 14 | 22787 | 25561 | 29245974 | -0.493 | -1.413 | 0.427 | 0.710 | 0.421 | 1.200 |
|  | Inappropriate antidiuretic | 10 | 22791 | 7712 | 29263823 | 0.690 | -0.417 | 1.796 | 1.614 | 0.868 | 3.001 |
|  | Thyrotoxic crisis | 10 | 22791 | 586 | 29270949 | 3.445 | **2.339** | 4.552 | 10.980 | **5.876** | 20.518 |
|  | Adrenal disorder | 9 | 22792 | 680 | 29270855 | 3.197 | **2.022** | 4.371 | 9.228 | **4.780** | 17.814 |
|  | Hypoparathyroidism | 9 | 22792 | 260 | 29271275 | 3.743 | **2.569** | 4.917 | 13.524 | **6.957** | 26.289 |
|  | Type 2 diabetes mellitus | 9 | 22792 | 20614 | 29250921 | -0.801 | -1.975 | 0.373 | 0.574 | 0.298 | 1.103 |
|  | Diabetes insipidus | 8 | 22793 | 927 | 29270608 | 2.791 | **1.536** | 4.047 | 6.957 | **3.468** | 13.954 |
|  | Pituitary enlargement | 8 | 22793 | 99 | 29271436 | 3.865 | **2.610** | 5.120 | 14.729 | **7.166** | 30.276 |
|  | Diabetes mellitus inadequate control | 6 | 22795 | 7422 | 29264113 | 0.049 | -1.434 | 1.533 | 1.035 | 0.465 | 2.304 |
|  | Diabetic metabolic decompen | 5 | 22796 | 1372 | 29270163 | 1.807 | **0.152** | 3.462 | 3.506 | **1.457** | 8.439 |
|  | Adrenalitis | 4 | 22797 | 33 | 29271502 | 3.089 | **1.189** | 4.989 | 8.560 | **3.032** | 24.164 |
|  | Hypogonadism | 4 | 22797 | 660 | 29270875 | 2.146 | **0.246** | 4.046 | 4.438 | **1.660** | 11.860 |
|  | Thyroiditis acute | 4 | 22797 | 237 | 29271298 | 2.710 | **0.810** | 4.610 | 6.573 | **2.447** | 17.661 |
|  | Diabetic coma | 3 | 22798 | 686 | 29270849 | 1.756 | -0.534 | 4.046 | 3.384 | **1.089** | 10.519 |
|  | Glucocorticoid deficiency | 3 | 22798 | 105 | 29271430 | 2.583 | **0.293** | 4.873 | 6.016 | **1.909** | 18.957 |
|  | Pituitary infarction | 3 | 22798 | 23 | 29271512 | 2.750 | **0.460** | 5.040 | 6.758 | **2.029** | 22.509 |
|  | Secondary hypogonadism | 3 | 22798 | 268 | 29271267 | 2.300 | **0.009** | 4.590 | 4.938 | **1.583** | 15.410 |
| Pembrolizumab+ ipilimumab | Fulminant type 1 diabetes mellitus | 17 | 733 | 538 | 29293048 | 5.089 | **4.260** | 5.918 | 34.082 | **20.917** | 55.534 |
|  | Type 1 diabetes mellitus | 14 | 736 | 3077 | 29290509 | 4.646 | **3.726** | 5.566 | 25.116 | **14.784** | 42.669 |
|  | Adrenal insufficiency | 9 | 741 | 4178 | 29289408 | 3.968 | **2.794** | 5.142 | 15.684 | **8.123** | 30.285 |
|  | Diabetic ketoacidosis | 9 | 741 | 17554 | 29276032 | 3.322 | **2.148** | 4.497 | 10.060 | **5.213** | 19.415 |
|  | Thyroiditis | 9 | 741 | 1604 | 29291982 | 4.133 | **2.959** | 5.308 | 17.574 | **9.091** | 33.971 |
|  | Hyperthyroidism | 8 | 742 | 6593 | 29286993 | 3.667 | **2.412** | 4.922 | 12.743 | **6.346** | 25.587 |
|  | Hypophysitis | 8 | 742 | 1675 | 29291911 | 3.968 | **2.713** | 5.223 | 15.670 | **7.795** | 31.503 |
|  | Hypothyroidism | 7 | 743 | 11719 | 29281867 | 3.228 | **1.873** | 4.584 | 9.406 | **4.468** | 19.804 |
|  | Diabetes mellitus | 4 | 746 | 28610 | 29264976 | 1.868 | -0.032 | 3.768 | 3.661 | **1.370** | 9.780 |
|  | Autoimmune hypothyroidism | 3 | 747 | 87 | 29293499 | 2.801 | **0.511** | 5.091 | 6.969 | **2.200** | 22.080 |
| Durvalumab+ tremelimumab | Adrenal insufficiency | 17 | 138 | 4170 | 29290011 | 5.067 | **4.238** | 5.896 | 33.677 | **20.330** | 55.786 |
|  | Hypopituitarism | 8 | 147 | 1009 | 29293172 | 4.072 | **2.817** | 5.327 | 16.830 | **8.239** | 34.377 |

* In **Table S3**, PT: preferred term; a: the number of records with endocrine AEs reported for ICIs; b: the number of records with any other AEs reported for ICIs; c: the number of records with any endocrine AEs for other drugs; d: the number of records reported other AEs for other drugs. IC_025_: the lower end of the 95% confidence interval of IC. IC_975_: the upper end of the 95% confidence interval of IC. IC_025_ greater than 0 was deemed a signal. ROR_025_: the lower end of the 95% confidence interval of ROR. ROR_975_: the upper end of the 95% confidence interval of IC. ROR_025_ exceeds 1 was deemed a signal. Bold text denotes a significant signal. Bold text denotes a significant signal. Only PTs with at least 3 records were listed.

**Table S4** PTs related to endocrine AEs after receiving ICIs in FAERS 2014 Q1-2019Q1, by descending frequency*

| PT | Frequency |
| --- | --- |
| Hypothyroidism | 885 |
| Adrenal Insufficiency | 730 |
| Hypophysitis | 688 |
| Hyperthyroidism | 472 |
| Diabetes Mellitus | 335 |
| Hyperglycaemia | 317 |
| Diabetic Ketoacidosis | 316 |
| Type 1 Diabetes Mellitus | 283 |
| Fulminant Type 1 Diabetes Mellitus | 224 |
| Thyroiditis | 218 |
| Hypopituitarism | 182 |
| Hypercalcaemia | 173 |
| Thyroid Disorder | 149 |
| Adrenocorticotropic Hormone Deficiency | 115 |
| Hypothalamo-Pituitary Disorder | 106 |
| Adrenal Disorder | 101 |
| Autoimmune Thyroiditis | 91 |
| Hypoglycaemia | 84 |
| Lymphocytic Hypophysitis | 76 |
| Secondary Adrenocortical Insufficiency | 72 |
| Type 2 Diabetes Mellitus | 53 |
| Endocrine Disorder | 52 |
| Adrenocortical Insufficiency Acute | 51 |
| Inappropriate Antidiuretic Hormone Secretion | 50 |
| Autoimmune Hypothyroidism | 40 |
| Diabetes Insipidus | 24 |
| Pituitary Enlargement | 23 |
| Diabetes Mellitus Inadequat | 21 |
| Gynaecomastia | 20 |
| Hypoparathyroidism | 18 |
| Goitre | 17 |
| Primary Hypothyroidism | 16 |
| Adrenalitis | 14 |
| Metastases to Adrenals | 13 |
| Thyrotoxic Crisis | 13 |
| Secondary Hypothyroidism | 12 |
| Silent Thyroiditis | 12 |
| Autoimmune Endocrine Disorder | 11 |
| Neuroendocrine Carcinoma of the Bladder | 11 |
| Diabetic Metabolic Decompensation | 10 |
| Glucocorticoid Deficiency | 9 |
| Hypogonadism | 9 |
| Endocrine Ophthalmopathy | 8 |
| Primary Adrenal Insufficiency | 8 |
| Hypoglycaemic Coma | 7 |
| Autoimmune Thyroid Disorder | 6 |
| Diabetic Nephropathy | 6 |
| Secondary Hypogonadism | 6 |
| Thyroid Cancer | 6 |
| Thyroiditis Acute | 6 |
| Adrenal Gland Cancer | 5 |
| Adrenal Neoplasm | 5 |
| Adrenomegaly | 5 |
| Exophthalmos | 5 |
| Hypercalcaemia Of Malignancy | 5 |
| Pituitary Haemorrhage | 5 |
| Thyroid Mass | 5 |
| Adrenal Mass | 4 |
| Diabetic Coma | 4 |
| Diabetic Hyperosmolar Coma | 4 |
| Infectious Thyroiditis | 4 |
| Polydipsia | 4 |
| Tetany | 4 |
| Androgen Deficiency | 3 |
| Diabetic Hyperglycaemic Coma | 3 |
| Diabetic Ketoacidotic Hyperglycaemic Coma | 3 |
| Hyperglycaemic Hyperosmolar Nonketotic Syndrome | 3 |
| Hyperparathyroidism | 3 |
| Hyperprolactinaemia | 3 |
| Latent Autoimmune Diabetes | 3 |
| Pituitary Infarction | 3 |
| Pituitary-dependent Cushing's syndrome | 3 |
| Hashitoxicosis | 2 |
| Thyroiditis Chronic | 2 |
| Polyglandular Disorder | 1 |

*In **Table S4**, PTs: preferred terms (PTs); FAERS: FDA Adverse Event Reporting System; Q1: first quarter.
